# Ischemic Postconditioning Alleviates Cerebral Ischemia–Reperfusion Injury Through Activating Autophagy During Early Reperfusion in Rats

**DOI:** 10.1007/s11064-018-2599-3

**Published:** 2018-07-25

**Authors:** Yameng Sun, Ting Zhang, Yan Zhang, Jinfeng Li, Lei Jin, Yinyi Sun, Nan Shi, Kangyong Liu, Xiaojiang Sun

**Affiliations:** 1 Department of Neurology, Shanghai Sixth People’s Hospital, Shanghai Jiao Tong University School of Medicine, Shanghai, 200233 People’s Republic of China; 2 Department of Neurology, Shanghai University of Medicine and Health Sciences Affiliated Zhoupu Hospital, Shanghai, 201318 People’s Republic of China

**Keywords:** Ischemic postconditioning, Autophagy flux, Cerebral ischemia–reperfusion injury, Mitochondrial dysfunction

## Abstract

This study aimed to investigate whether ischemic postconditioning (IpostC) alleviates cerebral ischemia/reperfusion (I/R) injury involved in autophagy. Adult Sprague–Dawley rats were divided into five groups: sham (sham surgery), I/R (middle cerebral artery occlusion [MCAO] for 100 min, then reperfusion), IpostC (MCAO for 100 min, reperfusion for 10 min, MCAO for 10 min, then reperfusion), IpostC+3MA (3-methyladenine, an autophagy inhibitor, administered 30 min before first reperfusion), and IpostC+Veh (vehicle control for IpostC+3MA group). Infarct volume was measured using cresyl violet staining. Autophagy-related proteins were detected by western blot and immunohistochemistry. Autophagosomes, autophagolysosomes, and mitochondrial damage were identified by transmission electron microscopy. Cortical cell apoptosis was detected by the TUNEL assay. Neurologic function was assessed using the modified Neurologic Severity Score. IpostC improved neurological function and reduced infarct volume after I/R (*P* < 0.05). These effects of IpostC were inhibited by 3MA (*P* < 0.05). Autophagosome formation was increased in the I/R and IpostC+Veh groups (*P* < 0.05), but not in the IpostC+3MA group. The I/R group showed enhanced LC3-II/LC3-I ratio, p62, and Cathepsin B levels and decreased LAMP-2 level (all *P* < 0.05 vs. sham), indicating dysfunction of autophagic clearance. IpostC reduced p62 and Cathepsin B levels and increased the LC3-II/LC3-I ratio, and nuclear translocation of transcription factor EB (all *P* < 0.05); these effects of IpostC were reversed by 3MA, suggesting IpostC enhanced autophagic flux. Furthermore, IpostC attenuated I/R-induced mitochondrial translocation of Bax and mitochondrial cytochrome-c release (all *P* < 0.05); 3MA inhibited these effects of IpostC (*P* < 0.05). In conclusion, IpostC may alleviate cerebral I/R injury by activating autophagy during early reperfusion.

**Electronic supplementary material:**

The online version of this article (10.1007/s11064-018-2599-3) contains supplementary material, which is available to authorized users.

## Introduction

Stroke is an acute disorder with serious complications and high mortality caused by impaired blood supply to the brain. Stroke is the fifth leading cause of death and the first cause of disability in adults [1]. The annual incidence of stroke is 795,000 in the USA, with an average of one victim every 40 s [1]. In China, the age-standardized incidence and prevalence of stroke are 1115/100,000 people and 247/100,000 person-years, with a mortality of 115/100,000 person-years [2]. Ischemic stroke accounts for 60–80% of all cases [2]. Despite an estimated cost of > 70 billion dollars per year, the treatment of stroke remains very limited and is mainly based on supportive therapies [1].

Restoration of blood supply during the reperfusion phase can induce ischemia–reperfusion (I/R) injury. Preconditioning is a brief subcritical ischemic episode that can mobilize intrinsic protective mechanisms against subsequent detrimental ischemia [3]. Since most cerebral ischemic events occur unpredictably, postconditioning (postC) could provide more therapeutic opportunities than preconditioning. Ischemic postC (IpostC) was first reported by Zhao et al. in 2006 [4]. IpostC is defined as a series of rapid intermittent interruptions of blood flow during reperfusion, and mobilizes the same protective mechanisms than preconditioning [3, 4]. Following acute ischemia, IpostC was found to decrease the necrotic area by 80% and reduce the extent of apoptosis [4, 5].

Autophagy is a process by which intracellular proteins and organelles are delivered to lysosomes by autophagosomes for degradation and recycling. Three types of autophagy are currently recognized: macroautophagy, microautophagy, and chaperone-mediated autophagy (CMA) [6]. Several key proteins in the autophagic cascade have been identified. Among them, Beclin-1 is essential for recruitment of autophagic proteins [7]. Markers of autophagic activity include the ratio of LC3-I (cytosolic) to LC3-II (membrane-bound), which correlates with autophagosome formation [8], whereas p62 levels are inversely correlated with autophagic activity [9]. LAMP-2 is another important protein involved in autophagy [10]. LAMP-2 deficiency causes autophagic vacuole accumulation in various tissues, resulting in cardiomyopathy and Danon disease in mice [11]. Accordingly, a recent study showed that chronic brain hypoperfusion in rats led to inefficient lysosomal clearance, which was caused by downregulation of LAMP-2 [12]. Cathepsin B belongs to the lysosomal cysteine protease family; it is upregulated in many pathological conditions, including ischemia, and its inactivation attenuates the ischemic apoptotic injury and autophagy [13–15]. Finally, under stress inducing autophagy, the bHLH-leucine zipper transcription factor EB (TFEB) translocates from the cytoplasm to the nucleus to regulate genes involved in autophagosome formation, autophagosome–lysosome fusion, and lysosomal exocytosis [16–19].

Several IpostC strategies have been demonstrated to activate autophagy in the ischemic brain [20–28]. Nevertheless, it remains unclear whether autophagy participates in the neuroprotective effects of IpostC in cerebral I/R [29–31]. Indeed, the roles of autophagy in IpostC are controversial. Some studies have reported that autophagy eliminates the neuroprotective effects of IpostC [22, 27, 32], whereas others have found that autophagy activation mimics IpostC-mediated neuroprotection [20, 26]. The apparent contradiction might be attributable to the complex nature of autophagy with regard to cargo selection [33]. In ischemic brains, the clearance of impaired mitochondria by autophagy is beneficial for neuronal survival, whereas nonselective autophagy is detrimental [31, 34]. Additional research is needed to better characterize the role of autophagy in the reperfusion phase after ischemia and its contribution to the final fate of neurons. Therefore, in the present study, we hypothesized that autophagy is a key process during IpostC and protects the ischemic brain.

## Methods and Materials

### Animals

All experimental procedures were approved by the Institutional Animal Care and Use Committee of Shanghai Jiao Tong University, Shanghai, China. Adult male Sprague–Dawley rats (9–10 weeks old, weighing 260–320 g) were obtained from the Shanghai Laboratory Animal Center, Shanghai, China. The rats were housed in individual cages at a constant room temperature with a 12-h light/dark cycle and were given free access to water and food. Every effort was made to minimize the number of rats used and animal suffering.

### Rat Model of Transient Middle Cerebral Artery Occlusion (MCAO) and Administration of Drugs

Transient focal cerebral ischemia and focal IpostC were carried out as previously described [35]. All surgical procedures were performed under an operating stereomicroscope, and body temperature (monitored by a rectal probe) was maintained at 37 ± 0.5 °C with a thermostatically controlled heating pad. Anesthesia was induced in a glass chamber using 4% isoflurane (mixed with 70% N_2_O and 30% O_2_) and maintained with a facemask using 2% isoflurane. A midline incision was made on the ventral neck surface, and the left common carotid artery (CCA), external carotid artery (ECA), and internal carotid artery (ICA) were isolated. A silicone-coated, round-tipped 4–0 monofilament nylon suture (Covidien, Mansfield, MA, USA) was gently introduced from the left CCA into the ICA through the stump of the ECA and advanced 20 ± 1 mm into the circle of Willis to occlude the branch point of the left middle cerebral artery (MCA). Reperfusion was performed 100 min after MCAO by withdrawal of the suture. Laser Doppler flowmetry (Moor Instruments, Devon, UK) was used to confirm ischemia in the area supplied by the left MCA. Animals without a cerebral blood flow reduction of at least 70% were excluded from the analysis, as were animals that died after induction of ischemia. The mortality in our study was less than 5%.

The autophagy inhibitor 3-methyladenine (3MA; M9281, Sigma-Aldrich, St. Louis, MO, USA) was administered by intracerebral ventricle (icv) injection; the dose (400 nM) was chosen according to a previous study [22]. Control rats received an equal volume of sterile saline. The anesthetized rat was placed on a stereotaxic apparatus, and 3MA or sterile saline was infused by a microinjector (1 µL/min) into the ventricular space ipsilateral to the ischemic region (− 0.92 mm anteroposterior, 1.5 mm mediolateral, and 3.5 mm dorsoventral to the bregma). Before each treatment, 3MA was freshly prepared in normal saline (final concentration, 100 nmol/µL) by heating the solution to 60–70 °C.

### Experimental Design

The animals were randomly assigned to five groups: sham group (surgical procedure undertaken but without MCAO); I/R group (MCAO for 100 min followed by reperfusion for 1, 6, 12, 24, or 48 h); IpostC group (MCAO for 100 min, reperfusion for 10 min, MCAO for a further 10 min, then reperfusion for 1, 6, 12, 24, or 48 h); IpostC+3MA group (MCAO for 100 min, reperfusion for 10 min with 400 nM of 3MA administered 30 min before reperfusion, MCAO for a further 10 min, then reperfusion for 24 h); and IpostC+Veh group (as for the IpostC+3MA group, but with an equal volume of sterile saline administered instead of 3MA). The experimental design and number of animals used are presented in Figure S1.

### Immunoblotting Analysis

The rats were anesthetized with an intraperitoneal injection of 10% chloral hydrate (350 mg/kg) and euthanized (by decapitation) after 1, 6, 12, 24, or 48 h of reperfusion (n = 3 rats/group/time point). The brains were removed on ice and the cerebral cortices ipsilateral to the ischemic area were dissected out rapidly and the tissues bordering the ischemic region were taken into RIPA lysis buffer (Millipore, Bedford, MA, USA) supplemented with 1 mmol/L phenylmethylsulfonyl fluoride (Thermo Fisher Scientific, Waltham, MA, USA) and a cocktail of protease inhibitors (Thermo Fisher Scientific). The tissues were sonicated and the protein concentration in the supernatant fraction was determined using a bicinchoninic acid assay (Thermo Fisher Scientific). The proteins (40 µg) were separated by electrophoresis on 8, 10, or 12% sodium dodecyl sulfate polyacrylamide gels and transferred onto 0.45-µm nitrocellulose membranes (Whatman, Piscataway, NJ, USA) or 0.2-µm polyvinylidene fluoride membranes (HVPPEAC12, Millipore). The membranes were blocked in 5% nonfat milk for 1 h and incubated overnight at 4 °C with primary antibodies against LC3 (1:1000, PM036, MBL, Nagoya, Japan), beclin-1 (1:400, sc-11427, Santa Cruz Biotechnology, Santa Cruz, CA, USA), p62 (1:1000, PM045, MBL), Bax (1:1000, #2772, Cell Signaling Technology, Danvers, MA, USA), cytochrome-c (1:1000, #11940, Cell Signaling Technology), β-actin (1:1000, sc-47778, Santa Cruz Biotechnology, Santa Cruz, CA, USA), LAMP-2 (1:200, sc-34245, Santa Cruz Biotechnology, Santa Cruz, CA, USA), TFEB (1:200, sc-48784, Santa Cruz Biotechnology, Santa Cruz, CA, USA), cytochrome-c oxidase subunit IV (COX IV; 1:1000, #4850, Cell Signaling Technology), cathepsin B (1:200, sc-365558, Santa Cruz Biotechnology, Santa Cruz, CA, USA) and H3 (1:1000, #9728, Cell Signaling Technology). The membranes were subsequently washed, incubated with horseradish peroxidase (HRP)-conjugated secondary antibodies for 1 h at room temperature and reacted with enhanced chemiluminescence substrate (Pierce, Rockford, IL, USA). The results were recorded using Quantity One imaging software (Bio-Rad, Hercules, CA, USA), and relative intensities were calculated using Gel-Pro Analyzer software (Media Cybernetics, Bethesda, MD, USA). For some experiments, subcellular fractionation was performed using a Nuclear and Cytoplasmic Protein Extraction Kit (p0027, Beyotime Biotechnology, Shanghai, China) [36, 37] and Tissue Mitochondria Isolation Kit (C3606, Beyotime Biotechnology) [38, 39].

### Transmission Electron Microscopy (TEM)

Cortical tissues bordering the ischemic core area from rats sacrificed at 24 h after ischemia (n = 3 rats/group) was fixed in 2.5% glutaraldehyde in 0.1 mol/L phosphate-buffered saline (PBS, pH 7.4) and cut by a vibratome into 50-µm-thick sections. The sections were postfixed with 1% osmium tetroxide for 1 h, dehydrated in a graded ethanol series (up to 100%) followed by dry acetone, and then embedded in Durcupan ACM Fluka (Sigma Aldrich, St Louis, MO, USA). The sections were further cut with a Reichert ultramicrotome (Leica, Wetzlar, Germany) into ultrathin (70 nm) sections that were stained with uranyl acetate and lead citrate and visualized using a CM-120 electron microscope (Philips, Amsterdam, Netherlands) [40].

For quantitative analyses of the numbers of autophagosomes, autolysosomes, and mitochondria in each group, 20 fields from each of three rats were examined using a protocol described previously [40]. Twenty-five randomly selected TEM images per animal were captured at a final magnification of 20,000×, and the number of autophagosomes and autolysosomes in each captured field was counted by visual inspection using previously established criteria for identification [7]. Autophagosomes were defined as double-membrane structures with an internal density similar to that of surrounding cytosol, while autolysosomes were considered to have only one limiting membrane and contained cytoplasmic material/organelles at various stages of degradation. For mitochondrial analysis, at least 20 randomly selected fields from tissues bordering the ischemic core area per animal were captured at a final magnification of 20,000×, and the number of mitochondria in each field from tissues surrounding the ischemic region was calculated by a technician blinded to the experimental design. The number of swollen mitochondria was also assessed. A mitochondrion was considered swollen when 5–6 times larger than normal mitochondria or in the presence of membrane rupture [41, 42].

### Immunohistochemistry

Each animal (n = 3 rats/group) was anesthetized 24 h after the surgical procedure and perfused transcardially with 4% paraformaldehyde in 0.1 mol/L PBS (pH 7.4). The brain was removed and postfixed for 24 h in 4% paraformaldehyde. Coronal sections bordering the ischemic core area were dehydrated in a graded ethanol series and then embedded in paraffin. Serial coronal sections (5 µm) were cut with a microtome, dewaxed in xylene, and rehydrated in graded alcohol. After incubation in PBS containing 0.3% Triton X-100 for 10 min, the mounted tissue sections underwent microwave antigen retrieval. To quench endogenous peroxidase activity, the sections were incubated in 0.3% H_2_O_2_ in methanol for 30 min, rinsed in PBS, and blocked in 1% horse serum (Vector Laboratories, Burlingame, CA, USA) dissolved in PBS for 60 min at room temperature. The tissues were subsequently incubated overnight at 4 °C with primary antibodies against LC3 (1:1000, L7543, Sigma-Aldrich, St Louis, Mo, USA), beclin-1 (1:1000, #3738, Cell Signaling Technology, Danvers, MA, USA), and p62 (1:1000, BML-PW9860, Enzo Life Sciences, Farmingdale, NY, USA). The sections were then incubated with biotinylated universal antibody (Vector Laboratories, Burlingame, CA, USA) for 60 min at room temperature, rinsed in PBS, incubated in Vectasta in ABC reagent for 60 min and rinsed again in PBS. The reaction product was visualized using 3,3′-diaminobenzidine (Vector Laboratories, Burlingame, CA, USA) until the desired intensity of staining was obtained. The sections were finally dehydrated in graded alcohol and mounted in Eukitt (Electron Microscopy Sciences, Hatfield, PA, USA) mounting medium. For each rat, five consecutive sections spaced at 200 µm were photographed for each of four regions of interest in the ipsilateral hemisphere, including the perifocal region in the cortex.

### Terminal Deoxynucleotidyl Transferase-Mediated dUTP Nick-end Labeling (TUNEL) Assay

The sections (from n = 3 rats/group, sacrificed at 24 h) were permeabilized with 0.4% Triton X-100 for 10 min and incubated with 10% normal donkey serum for 1 h at room temperature followed by anti-NeuN antibody (1:200, MAB377, Millipore corp., Billerica, MA, USA) overnight at 4 °C. The tissues were washed (0.1% Triton X-100 for 3–10 min), incubated with donkey anti-mouse Alexa Fluor 594-conjugated secondary antibody at room temperature for 2 h, and then washed again (PBS for 3–10 min). TUNEL staining was performed using an in situ cell death detection kit (Roche Applied Science, Nonnenwald, Germany), in accordance with the manufacturer’s instructions. The mounted sections were incubated for 2 min in a freshly prepared aqueous solution of 0.1% Triton X-100 and 0.1% sodium citrate, immersed in 50 µL of TUNEL mixture for 1 h at 37 °C, rinsed in PBS, and mounted with FluorSave. For peroxidase revelation of TUNEL, sections were incubated for 2 h at room temperature with an anti-FITC antibody conjugated with biotin (Sigma-Aldrich, St Louis, MO, USA). The sections were viewed and analyzed using laser scanning confocal microscopy (LSCM, FV1000, Olympus, Tokyo, Japan) and a digital imaging software (FV10-ASW 1.5 Viewer). The number of NeuN-positive or TUNEL-positive neurons per 200-µm length of cortical tissue bordering the ischemic core area were counted in five sections per animal. Cell counts on each of the five sections were averaged to provide the mean value. The mean value was used for analysis.

### Neurobehavioral Assessments

Neurologic function (n = 9 rats/group) was assessed 24 h after reperfusion by an experimenter blinded to the animal grouping. A modified Neurologic Severity Score (mNSS) ranging 0–14 was adopted. The mNSS assessment included raising the rat by the tail (0–3), walking on the floor (0–3), beam balance tests (0–6), and absence of reflexes (0–2) [43].

### Measurements of Infarct Volume

Infarct volume was determined using cresyl violet staining (from n = 6 rats/group, sacrificed at 24 h). A series of 20-µm-thick coronal sections from the anterior commissure to the hippocampus was obtained, and the first of every 10 consecutive sections were mounted on slides (i.e. a distance between adjacent sections on the slides of 200 µm), for a total of 18–25 sections. The frozen sections were stained with cresyl violet (Sigma-Aldrich, St Louis, MO, USA), and the ischemic area within each section was delineated using an image analysis software (ImageJ, National Institutes of Health, Bethesda, MD, USA). The infarct volume between two adjacent sections was calculated using the formula: ${1 \mathord{\left/ {\vphantom {1 3}} \right. \kern-0pt} 3}\, \times \,{\text{h}}\, \times \,\left[ {{\text{S1}}\,+\,{\text{S2}}\,+\,\sqrt {\left( {{\text{S1}}\, \times \,{\text{S2}}} \right)} } \right]$, where S1 and S2 are the infarct areas of the two sections. Infarct volume was derived from the sum of all infarct volumes between adjacent sections.

### Statistical Analysis

**Fig. 1:**
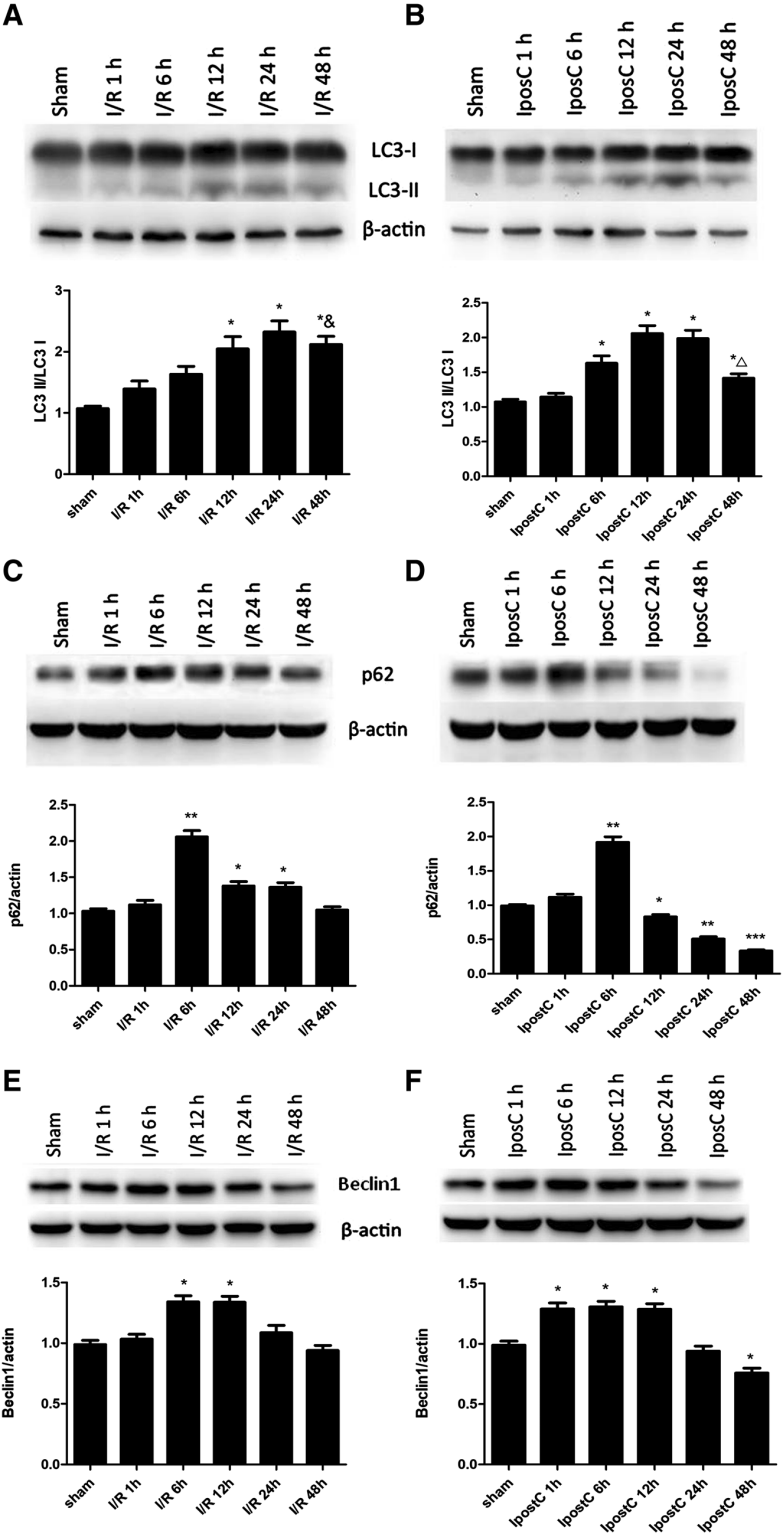
Effects of ischemic postconditioning (IpostC) on time-dependent expression levels of LC3, p62, and Beclin 1 in the cerebral cortex following ischemia–reperfusion (I/R) injury. Rats were subjected to cerebral I/R injury with or without IpostC, and this was followed by reperfusion for 1, 6, 12, 24, or 48 h. LC3, p62, and Beclin 1 protein expression levels in cerebral cortex were determined by western blotting. **a** LC3 expression at different times after reperfusion in the I/R group. **b** LC3 expression at different times after reperfusion in the IpostC group. **c** p62 expression at different times after reperfusion in the I/R group. **d** p62 expression at different times after reperfusion in the IpostC group. **e** Beclin 1 expression at different times after reperfusion in the I/R group. **f** Beclin 1 expression at different times after reperfusion in the IpostC group. Data presented as the mean ± standard error of the mean (SEM) (n = 3 each group). *P < 0.05, **P < 0.01, ***P < 0.001 vs. sham group; ^△^*P* < 0.05 vs. IpostC 24 h; ^&^*P* > 0.05 vs. IpostC 24 h

**Fig. 2:**
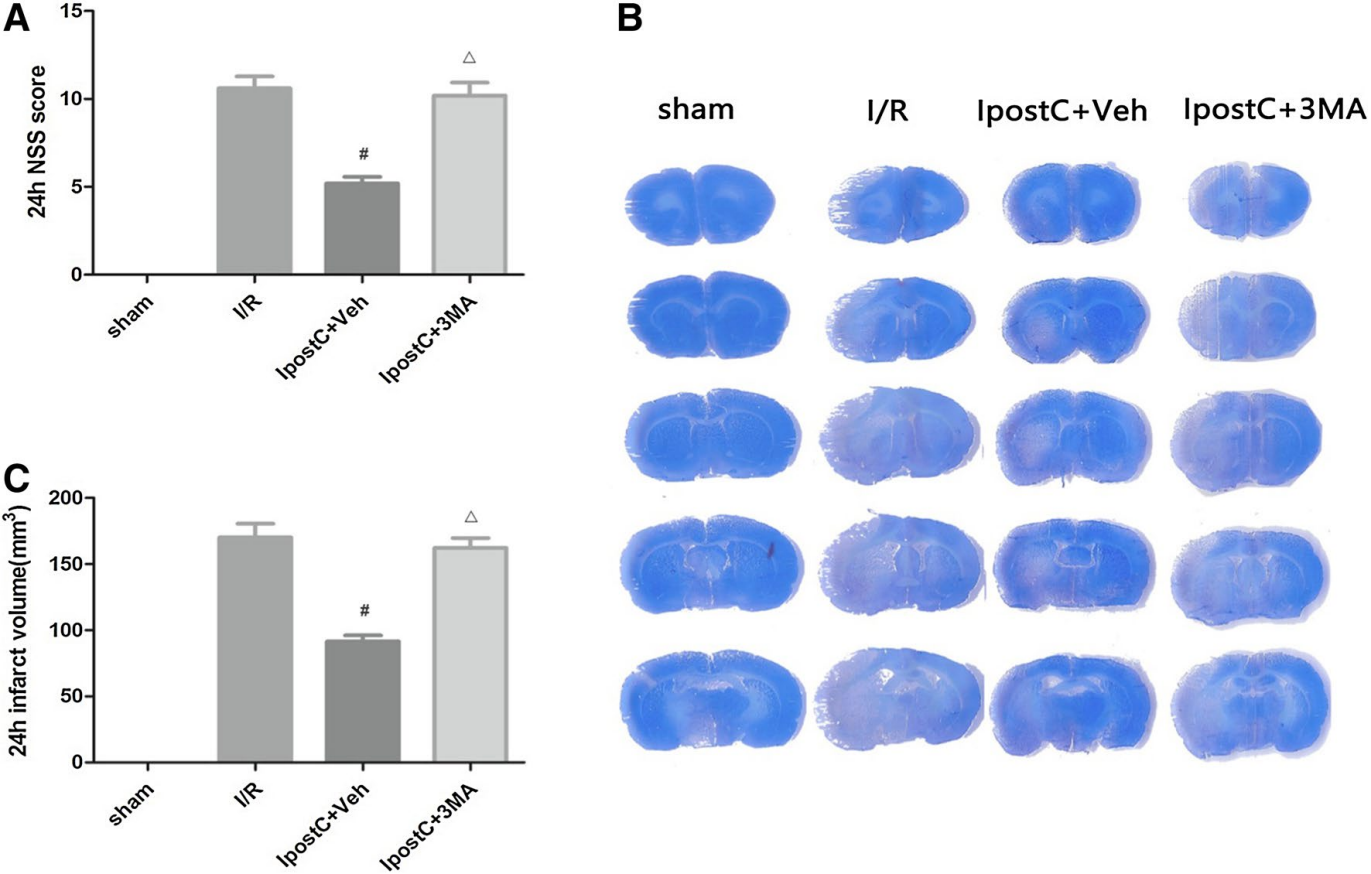
An inhibitor of autophagy attenuated the protective effect of IpostC against cerebral I/R injury. **a** Neurologic function assessed using the modified Neurologic Severity Score (mNSS) in the sham, I/R, IpostC+Veh and IpostC+3-methyladenine (3MA) groups. Data presented as the mean ± SD (n = 9 each group). **b** Representative images of brain slices stained with cresyl violet in different groups. Regions of cerebral infarction are stained white. **c** Infarct volume quantified using ImageJ software. Data presented as the mean ± standard deviation (SD) (n = 6 each group). ^#^*P* < 0.05 vs. I/R group; ^△^*P* < 0.05 vs. IpostC+Veh group

**Fig. 3:**
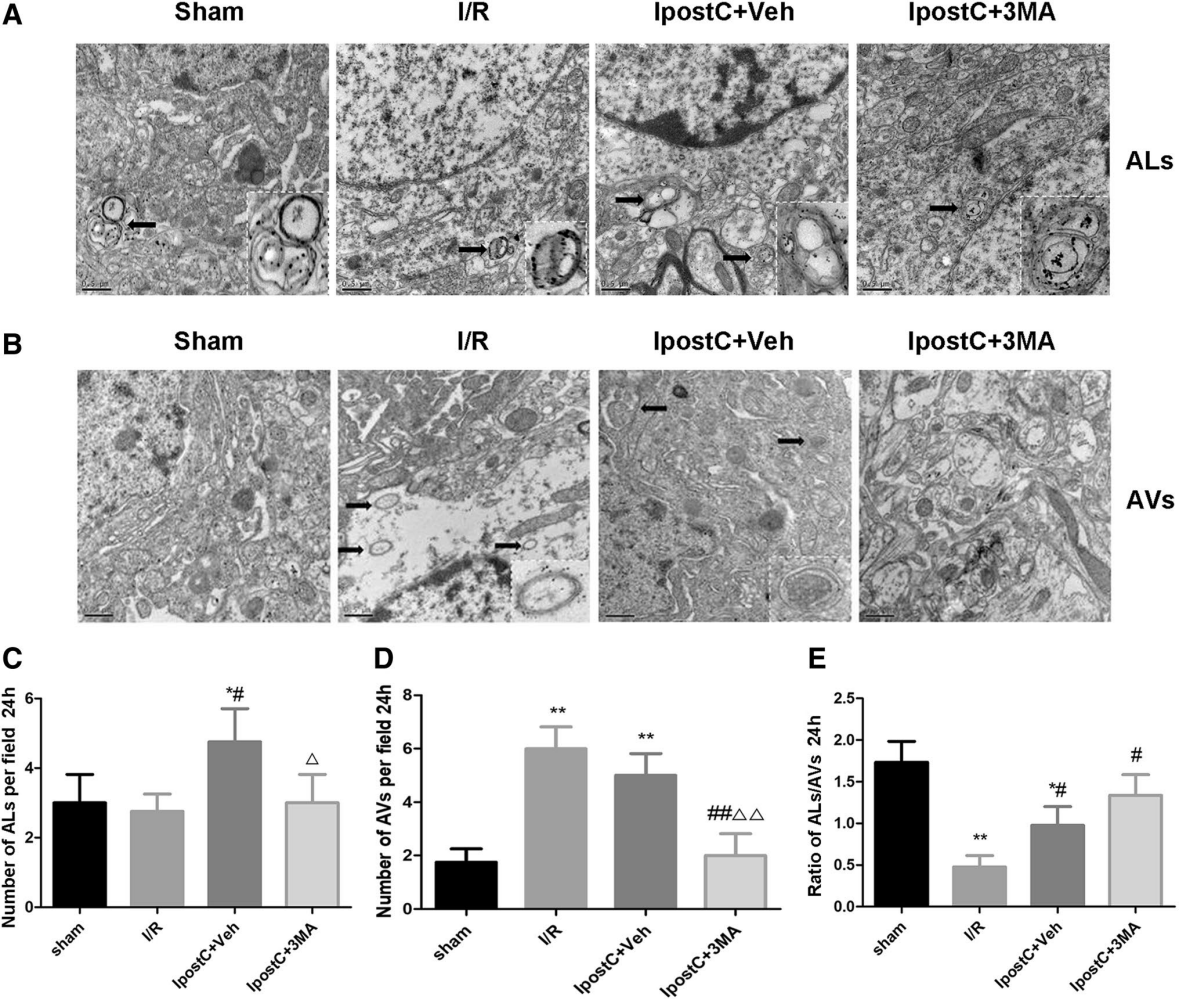
Effects of IpostC on formation of autophagosomes and autolysosomes in the cerebral cortex following I/R injury. **a** Representative transmission electron microscopy images of autolysosomes (ALs) in the various groups. Black arrows indicate ALs. **b** Representative transmission electron microscopy images of autophagosomes (AVs) in the various groups. Black arrows indicate AVs. Scale bars: 0.5 µm. Insets in the pictures show enlarged autolysosomes or autophagosomes which were indicated by black arrows. **c** Quantitative analysis of the number of ALs per field in the various groups. **d** Quantitative analysis of the number of AVs per field in the various groups. **e** Quantitative analysis of the AL/AV ratio in the various groups. Data presented as the mean ± SEM (n = 3 each group). *P < 0.05, **P < 0.01 vs. sham group; ^#^*P* < 0.05, ^##^*P* < 0.01 vs. I/R group; ^△^*P* < 0.05, ^△△^*P* < 0.01 vs. IpostC+Veh group

**Fig. 4:**
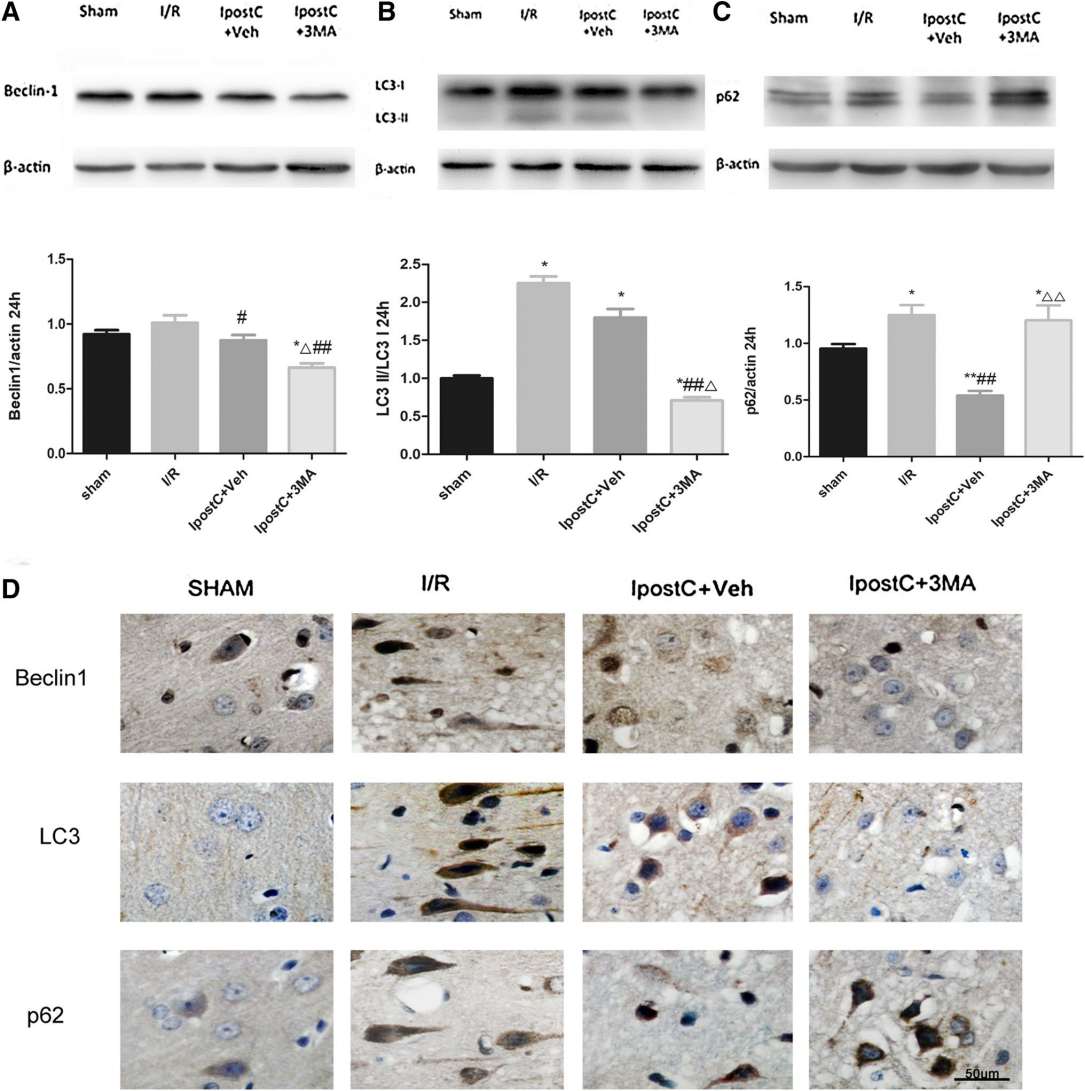
Effects of IpostC on expression levels of autophagy-related proteins (beclin-1, LC3, p62) in the cerebral cortex following I/R injury. Western blot was used to determine the beclin-1 (**a**), LC3-II/LC3-I ratio (**b**) and p62 (**c**) protein expression levels in the various groups. **d** Representative immunohistochemical images showing the localization of beclin-1, LC3, and p62 in the cerebral cortex of the hemisphere ipsilateral to the ischemic region. Scale bar: 50 µm. Data presented as the mean ± SEM (n = 3 each group). *P < 0.05, **P < 0.01 vs. sham group; ^#^*P* < 0.05, ^##^*P* < 0.01 vs. I/R group; ^△^*P* < 0.05, ^△△^*P* < 0.01 vs. IpostC+Veh group

**Fig. 5:**
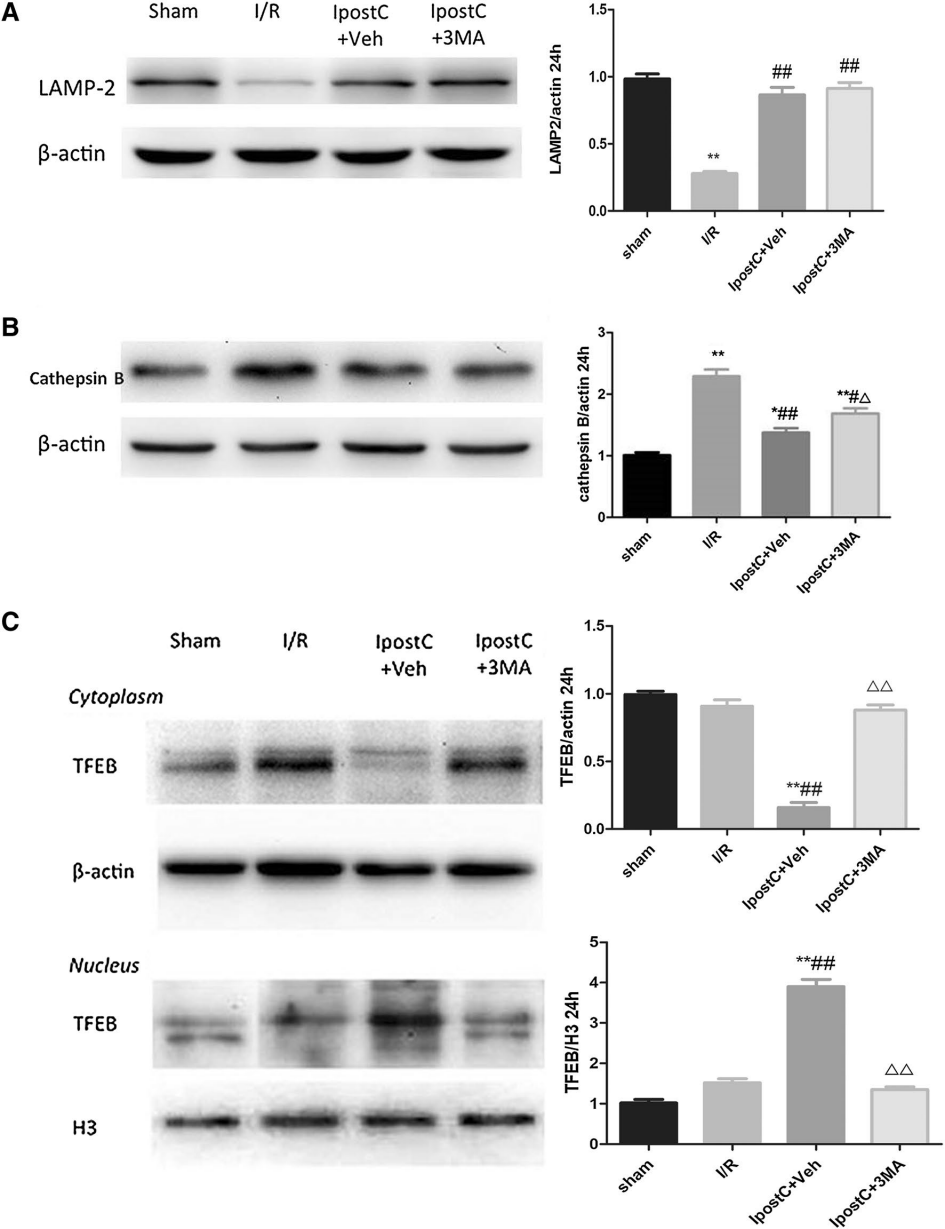
Effects of IpostC on expression levels of autophagy-related proteins (LAMP-2, Cathepsin B and TFEB) in the cerebral cortex following I/R injury. LAMP-2 (**a**) and Cathepsin B (**b**) protein expression levels in the various groups were determined by western blot. **c** Subcellular fractionation of the cerebral cortex was employed to determine the subcellular location of TFEB by Western blot. Data presented as the mean ± SEM (n = 3 each group). *P < 0.05, **P < 0.01 vs. sham group; ^#^*P* < 0.05, ^##^*P* < 0.01 vs. I/R group; ^△^*P* < 0.05, ^△△^*P* < 0.01 vs. IpostC+Veh group

**Fig. 6:**
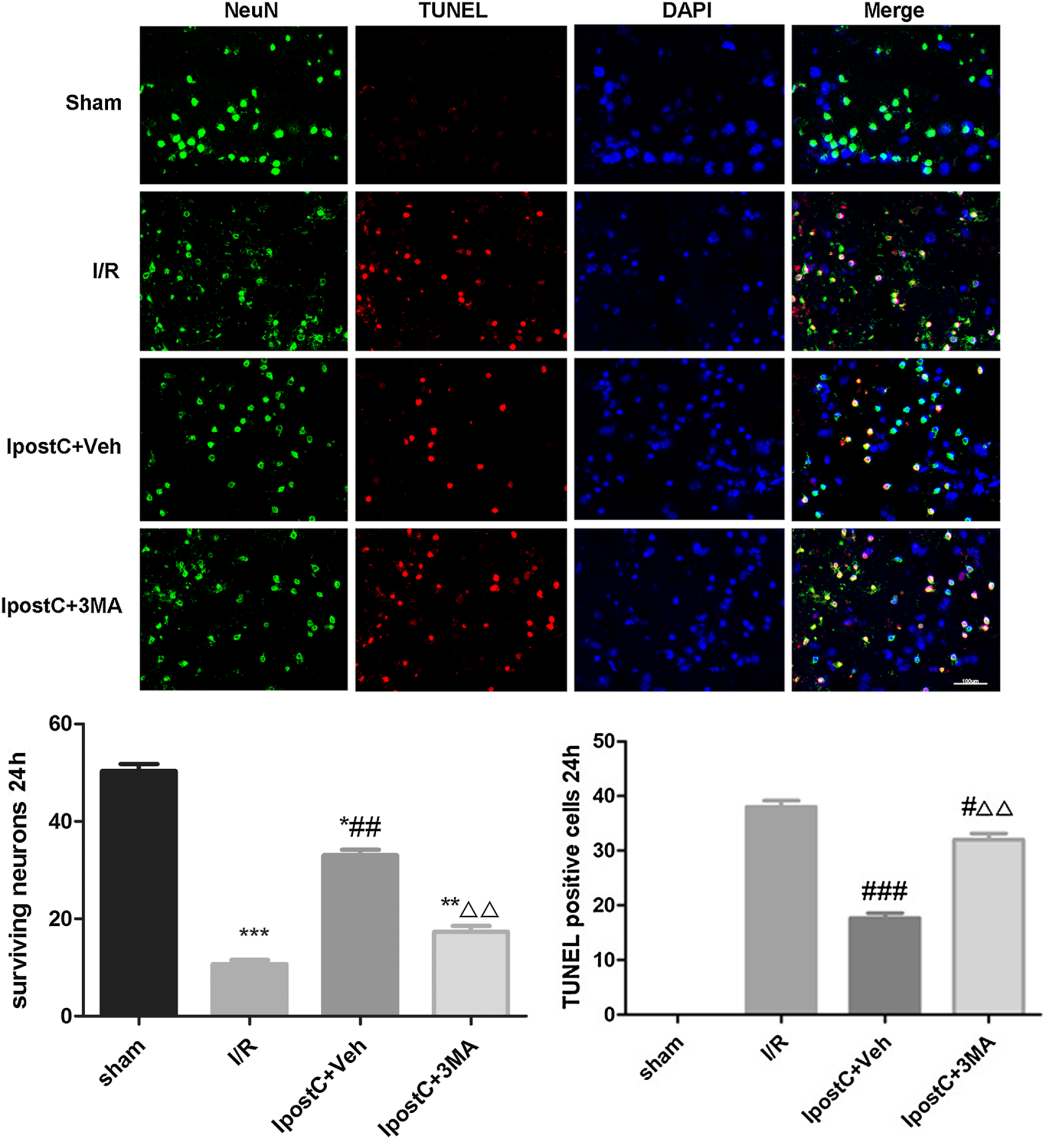
Effect of IpostC on I/R-induced apoptosis in the cerebral cortex. Representative images showing immunofluorescent staining for the neuronal marker NeuN (green) and the TUNEL assay (red) in sections of the cerebral cortex. DAPI (blue): nucleus. Magnification: ×40. Scale bar: 100 µm. Data presented as the mean ± SEM (n = 3 each group). *P < 0.05, **P < 0.01, ***P < 0.001 vs. sham group; ^#^*P* < 0.05, ^##^*P* < 0.01, ^###^*P* < 0.001 vs. I/R group; ^△△^*P* < 0.01 vs. IpostC+Veh group

**Fig. 7:**
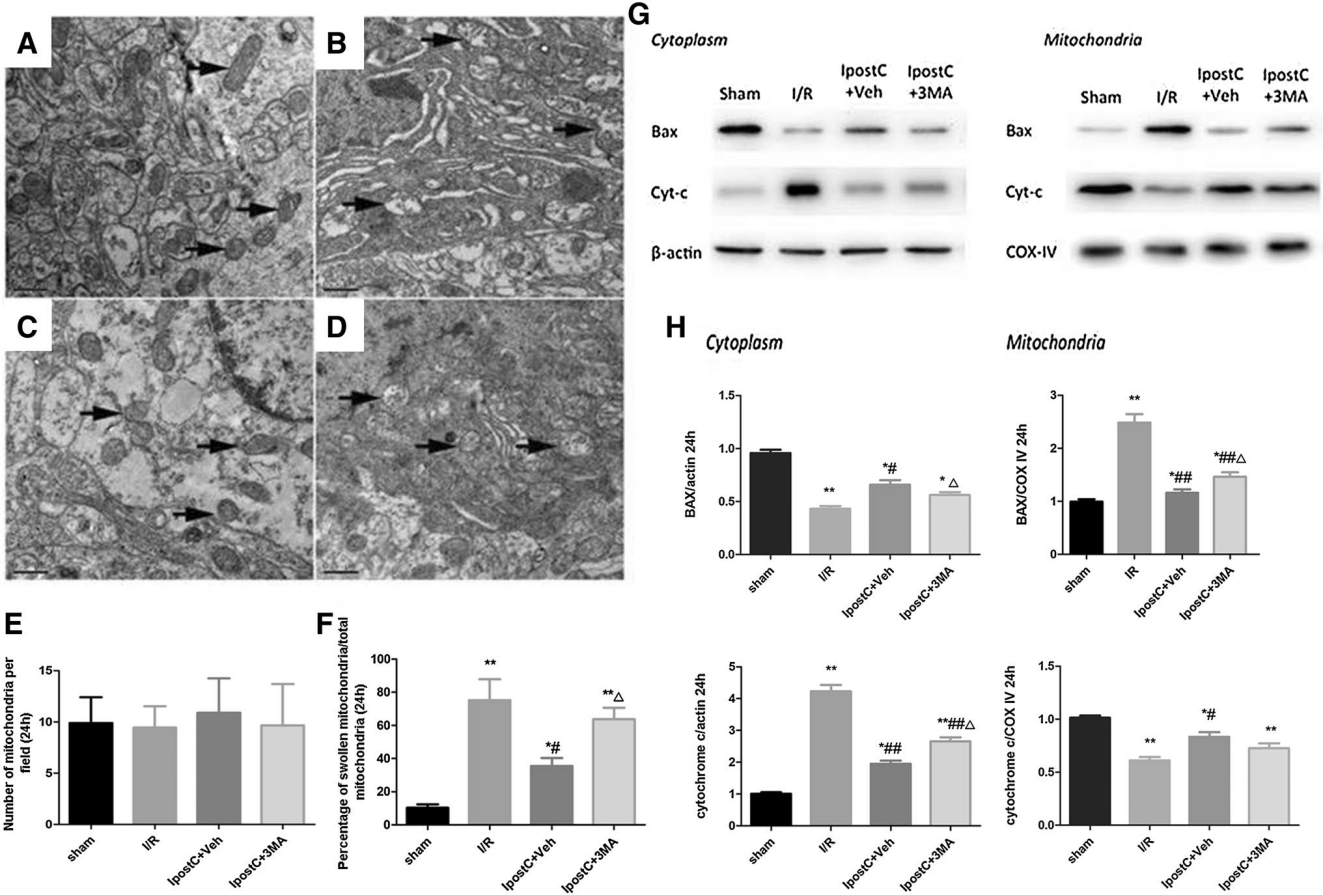
Effect of IpostC on mitochondrial number and morphology and mitochondria-dependent apoptosis related protein (Bax and cytochrome-c) expression levels in the cerebral cortex following I/R injury. (**a**–**d**) Representative transmission electron microscopy images to show mitochondrial morphology in the sham (**a**), I/R (**b**), IpostC+Veh (**c**) and IpostC+3MA (**d**) groups. Mitochondrial vacuoles are indicated by arrows. Scale bars: 0.5 µm. **e** Quantitative analysis of the number of mitochondria per field. **f** Quantitative analysis of the ratio of swollen mitochondria to total mitochondria. **g** Representative immunoblots showing the distribution of Bax and cytochrome-c in the cytoplasm and mitochondria in the various groups, determined after subcellular fractionation had been performed. **h** Quantitative analysis of the cytoplasmic and mitochondrial levels of Bax and cytochrome-c. Data presented as the mean ± SEM (n = 3 each group). *P < 0.05, **P < 0.01 vs. sham group; ^#^*P* < 0.05, ^##^*P* < 0.01 vs. I/R group; ^△^*P* < 0.05 vs. IpostC+Veh group

Statistical analysis was carried out using Prism 5 software (GraphPad Software, San Diego, CA, USA). Data are presented as mean ± standard deviation (SD) (for mNSS score and infarct volume) or mean ± standard error of the mean (SEM) (for western blot and TEM). Comparisons among multiple groups were made using one-way (Figs. 2, 3, 4, 5, 6, 7) or two-way (Fig. 1) analysis of variance (ANOVA) followed by the Tukey’s post-hoc test. *P* < 0.05 was considered statistically significant.

## Results

### Effects of IpostC on Time-Dependent Expression Levels of LC3, p62 and Beclin-1 in the Cerebral Cortex Following Cerebral I/R Injury

To determine the extent of autophagy activation in the MCAO model of focal cerebral ischemia, the cortical expressions of three autophagic markers, LC3, p62 and beclin-1, were determined at 1, 6, 12, 24, or 48 h post-ischemia (Fig. 1). Western blot revealed that the LC3-II/LC3-I ratio in the I/R group started to raise by 6 h, reaching significantly high levels at 12 h (*P* < 0.05 vs. sham group), peaked at 24 h (*P* < 0.05 vs. sham group) and was maintained at a high level at 48 h post-ischemia (*P* < 0.05 vs. sham group; *P* > 0.05 vs. I/R group at 24 h, Fig. 1a). The expression of p62 in the I/R group also increased significantly to peak at 6 h (*P* < 0.01 vs. sham group). The p62 levels subsequently decreased but remained higher than that in the sham group at 12 and 24 h (both *P* < 0.05 vs. sham group), before returning to baseline levels at 48 h (Fig. 1c). Furthermore, the expression of beclin-1 in the I/R group peaked at 6 h (*P* < 0.05 vs. sham group). The beclin-1 levels subsequently returned to baseline levels by 24 h (both *P* > 0.05 vs. sham group) (Fig. 1e).

In the IpostC group, the LC3-II/LC3-I ratio started to increase at 6 h (*P* < 0.05 vs. sham group), peaked at 12 h (*P* < 0.05 vs. sham group), but was not fully maintained at 48 h post-ischemia (*P* < 0.05 vs. IpostC 24 h) (Fig. 1b). As shown in Fig. 1d, p62 expression in the IpostC group also peaked at 6 h (*P* < 0.01 vs. sham group), but the expression of p62 subsequently decreased progressively to reach a level at 48 h that was significantly lower than that in the sham group (*P* < 0.001; Fig. 1d). The fall in the p62 levels in the IpostC group is consistent with increased autophagic degradation of p62 resulting from an enhancement of autophagy by IpostC. In addition, the expression of beclin-1 in the IpostC group peaked at 1 h and remained high at 6 and 12 h (*P* < 0.05 vs. sham group). The beclin-1 levels subsequently returned to baseline levels at 24 h (*P* > 0.05 vs. sham group) and reached lower values than in the control group at 48 h (*P* < 0.05 vs. sham group) (Fig. 1f).

### An Inhibitor of Autophagy Attenuated the Protective Effect of IpostC Against Cerebral I/R injury

mNSS score after 24 h of reperfusion was significantly lower in the IpostC+Veh group than in the I/R group or IpostC+3MA group (both *P* < 0.05, Fig. 2a). Furthermore, staining of brain sections with cresyl violet revealed areas of cerebral infarction (stained white) in the I/R, IpostC+3MA, and IpostC+Veh groups but not in the sham group (Fig. 2b). Quantification of the staining showed that the infarct volume in the IpostC+Veh group was significantly lower than that in the I/R group or IpostC+3MA group (both *P* < 0.05, Fig. 2c).

### Effects of IpostC on Formation of Autophagosomes and Autolysosomes in the Cerebral Cortex Following I/R Injury

TEM was used to observe autophagosomes and autolysosomes in order to monitor the induction of autophagy (Fig. 3). Compared with the sham and IpostC+3MA groups, the number of autolysosomes per field was significantly higher in the IpostC+Veh group (*P* < 0.05 vs. sham group, I/R group, and IpostC+3MA group) (Fig. 3c). Compared with the sham and the IpostC+3MA groups, the number of autophagosomes per field was significantly higher in both the I/R and IpostC+Veh groups (both *P* < 0.01) (Fig. 3d). Similarly, the ratio of autolysosomes to autophagosomes was significantly lower in the I/R group and IpostC+Veh group than in the sham and the IpostC+3MA groups (all *P* < 0.05). The autolysosome to autophagosome ratio was slightly higher in the IpostC+Veh group than in the I/R group (*P* < 0.05; Fig. 3e). Notably, the ratio of autolysosomes to autophagosomes in the IpostC+3MA group was significantly higher than that in the I/R group (*P* < 0.05) (Fig. 3e).

### Effects of IpostC on Expression Levels of Autophagy-Related Proteins in the Cerebral Cortex Following I/R Injury

Western blotting (Fig. 4) revealed that cerebral I/R upregulated the levels of autophagy-related proteins (LC3, beclin-1, and p62) in the cerebral cortex. IpostC partially reversed the increased levels of beclin-1 (*P* < 0.05) and p62 (*P* < 0.01) at 24 h after I/R (Fig. 4a, c), but not LC3-II/LC3-I (*P* > 0.05) (Fig. 4b). Furthermore, the expressions of beclin-1 and LC3 were lower in the IpostC+3MA group than in the IpostC+Veh group (both *P* < 0.05) (Fig. 4a, b), whereas that of p62 (which is degraded by autophagosomes) was higher (*P* < 0.01) (Fig. 4c), consistent with the inhibition of autophagy by 3MA. These results were supported by the immunohistochemistry experiment (Fig. 4d).

The protein expression levels of LAMP-2, cathepsin B, and TFEB were further determined by western blot. The LAMP-2 protein levels were significantly lower in the I/R group than in the sham group at 24 h post-ischemia (*P* < 0.01; Fig. 5a). The I/R-induced downregulation of LAMP-2 was not observed in the IpostC+Veh and the IpostC+3MA groups (*P* > 0.05) (Fig. 5a), indicating that IpostC attenuated I/R-induced LAMP-2 downregulation by enhancing autophagy. The cathepsin B protein levels were significantly higher in the I/R group than in the sham group at 24 h post-ischemia (*P* < 0.01; Fig. 5b). The I/R-induced upregulation of cathepsin B was observed in the IpostC+Veh and the IpostC+3MA groups (*P* < 0.05 vs. sham group), but the levels were lower than in the I/R group (*P* < 0.05) (Fig. 5b). Subcellular fractionation experiments were performed to address whether IpostC and autophagy were linked to nuclear translocation of TFEB. There was a significant increase in nuclear TFEB after IpostC treatment that was blocked by the administration of 3MA (*P* < 0.01; Fig. 5c).

### Effect of IpostC on I/R-Induced Apoptosis in the Cerebral Cortex

TUNEL assay showed that I/R induced the apoptosis of neurons in the cerebral cortex (Fig. 6). IpostC attenuated I/R-induced neuronal apoptosis (*P* < 0.001) and 3MA inhibited this effect of IpostC (*P* < 0.01) (Fig. 6).

Mitochondrial number and morphology were also assessed using TEM (Fig. 7a–d). The total number of mitochondria did not differ significantly among the four groups (Fig. 7e), but the ratio of swollen mitochondria to total mitochondria was significantly higher in the I/R (*P* < 0.01), IpostC+Veh (*P* < 0.05), and IpostC+3MA (*P* < 0.01) groups than in the sham group (Fig. 7f). The ratio of swollen mitochondria to total mitochondria in the IpostC+Veh group was significantly lower than that in both the I/R and IpostC+3MA groups (both *P* < 0.05; Fig. 7f).

To explore whether IpostC and autophagy were associated with mitochondrial translocation of Bax, subcellular fractionation experiments were performed (Fig. 7g). The mitochondrial Bax levels were increased significantly after I/R (*P* < 0.01), and IpostC attenuated mitochondrial translocation of Bax, whereas 3MA inhibited this effect of IpostC (*P* < 0.05; Fig. 7h).

Mitochondrial translocation of cytochrome-c was also shown by subcellular fractionation experiments (Fig. 7g). The cytoplasmic cytochrome-c levels were increased in the I/R group compared with the sham group (*P* < 0.01; Fig. 7h), indicating mitochondrial dysfunction. Mitochondrial release of cytochrome-c after I/R was reduced by IpostC (*P* < 0.01; Fig. 7h), while 3MA attenuated this effect of IpostC (*P* < 0.05; Fig. 7h).

## Discussion

Stroke is associated with high morbidity and mortality, highlighting the need for novel clinical approaches to treating this disease. IpostC applied at the onset of reperfusion has been reported to ameliorate I/R injury [44]. Multiple mechanisms have been suggested to contribute to the neuroprotective mechanisms of IpostC, including regulation of synaptic signaling, attenuation of oxidative stress and inflammation, maintenance of mitochondrial integrity, inhibition of endoplasmic reticulum stress, activation of the phosphoinositide 3-kinase/Akt pathway, inhibition of apoptosis and protection of the neurovascular unit [21–25, 27, 28]. Nevertheless, it is unclear whether autophagy during early reperfusion is influenced by IpostC or plays a role in the effects of IpostC. The results of the present study strongly suggest that IpostC improved neurologic function and reduced infarct volume after I/R injury. These effects of IpostC were inhibited by 3MA, an inhibitor of autophagy. Indeed, autophagosome formation was increased in the I/R and IpostC+Veh groups, but not in the IpostC+3MA group. These results support the hypothesis stating that autophagy participates in the protective effect of IpostC on I/R injury, as supported by previous studies performed in different models and organs [21–25, 27, 28].

Compared with the sham group, the I/R group showed an increased number of autophagosomes, higher levels of p62, and lower levels of LAMP-2. These findings are consistent with dysfunctional autophagic flux and clearance during the reperfusion period, possibly due to impaired fusion of autophagosomes with lysosomes or impaired autolysosomal degradation. IpostC significantly reduced infarct volume and improved neurologic function, and these effects were attenuated by 3MA (an inhibitor of autophagy), suggesting that activation of autophagy was involved in the beneficial effects of IpostC. Furthermore, the low levels of the p62 protein, high levels of the LAMP-2 protein, and nuclear translocation of TFEB in the IpostC group suggested that IpostC promoted autophagic flux that progressed to its completion. In addition, cathepsin B levels were higher in the I/R group, while the increase was attenuated by IpostC. This is supported by previous studies showed that cathepsin B is involved in autophagy [15], and that inhibition of cathepsin B contributed to neuroprotection against cerebral ischemic injury [14]. The detection of apoptosis-related proteins revealed that IpostC prevented the mitochondrial translocation of Bax and reduced cytochrome-c release from mitochondria associated with I/R. Moreover, 3MA inhibited these effects of IpostC. Taken together, these results strongly suggest that the autophagic pathway following I/R injury may be dysfunctional and that IpostC ameliorates cerebral I/R injury through activation of autophagy.

Autophagic flux encompasses the entire process of autophagy, including the formation of autophagosomes, the transport of substrates to lysosomes, the degradation of substrates, and the release of macromolecules to the cytoplasm [45]. Therefore, it is necessary to monitor autophagic flux in order to detect autophagic activity [45]. Membrane-bound LC3-II can be used as a marker of autophagy because it is retained in mature autophagosomes and is not released until fusion with the lysosome [46]. p62 participates in the selective degradation of ubiquitinated proteins by autophagy [47], thus the expression of p62 directly reflects the level of autophagic clearance. The levels of both LC3-II and p62 proteins need to be measured in order to adequately monitor the autophagic flux [48].

Previous research indicated that ischemic stroke might lead to the activation of autophagy through mechanisms that include a depletion of nutrients, oxidative stress, and protein misfolding [49]. The activation of autophagy after ischemia has been detected in models of focal cerebral ischemia [50] and global cerebral ischemia [51]. Wei et al. [28] observed that IpostC activated autophagy and inhibited myocardial cell apoptosis in rat hearts subjected to I/R injury. Hao et al. [24] reported that 3MA suppressed the cardioprotective effects of IpostC against I/R injury and suggested that IpostC promoted autophagy partially via activation of the neuronal nitric oxide synthase/adenosine monophosphate-activated protein kinase (AMPK)/mechanistic target of rapamycin (mTOR) pathway. Consistent with the aforementioned study, Guo et al. [23] highlighted the crucial role of autophagy in the protective mechanisms of IpostC in cardiomyocytes, through the regulation of beclin-1 and AMPK/mTOR signaling pathways. Using the transient MCAO model of cerebral I/R, Qi et al. [25, 26] found that remote limb IpostC activated autophagy and reduced cell death through the AKT/GSK3β pathway and Bcl-2 phosphorylation, and the authors suggested that this may contribute to the attenuation of mitochondrial damage by IpostC. Zhang et al. [31] reported that inhibition of autophagy during the reperfusion phase worsened neuronal death in an in vivo model (MCAO in mice) and an in vitro model (cultured cortical neurons) of I/R.

The protective role of autophagy was attributed to mitophagy-related mitochondrial clearance (possibly involving the E3 ligase, PARK2) and inhibition of downstream apoptosis. On the other hand, a published study yielded contradictory results. Indeed, Gao et al. [22] created a focal cerebral ischemia model involving permanent distal MCAO plus transient bilateral CCA occlusion and reported that IpostC inhibited the upregulation of LC3/beclin-1 and reversed the reduction of p62 caused by ischemia. Furthermore, rapamycin (an inducer of autophagy) attenuated the effects of IpostC while 3MA induced neuroprotection [22]. We suggest that two important factors might contribute to the discrepancies between the latter study and ours. First, we performed IpostC using a different experimental method. Second, the markers detected in this study were different from those used in the study of Gao et al. [22], in order to more clearly delineate changes in the autophagy pathway. After formation, autophagosomes fuse with lysosomes for subsequent clearance [22]. Impaired clearance of autophagosomes could result in their accumulation, which could be incorrectly interpreted as enhanced autophagy. Therefore, we detected lysosomal activity by measuring the expression of LAMP-2, a major protein on the lysosomal membrane that plays an important role in the fusion of autophagosomes and lysosomes [18]. In addition, we measured the nuclear translocation of TFEB protein, which regulates lysosomal biogenesis and autophagy. TFEB promotes the autophagosomal-lysosomal fusion for protein clearance and prevents accumulation of autophagosomes [52, 53]. TFEB can upregulate the expression of nearly two-thirds of autophagy–lysosome genes and its overexpression shows potential therapeutic effects in cardiovascular disease by rescuing lipid-induced lysosomal dysfunction [16, 52, 54] as well as enhancing lipolysis [55]. Normally, TFEB is located in the cytosol and on the lysosomal surface, where it interacts with mTOR in its inactive phosphorylated form; however, in response to stimuli, TFEB translocates to the nucleus [56].

Our observations indicate that IpostC can stimulate functional autophagy that protects against cerebral I/R injury, and that the underlying mechanism may involve translocation of TFEB to the nucleus and transcription of target genes that promote autophagic flux. The clearance of impaired mitochondria by autophagy is beneficial for neuronal survival, as previously shown [25, 34]. The ratio of swollen mitochondria to total mitochondria in the IpostC+Veh group was significantly lower than that in both I/R and IpostC+3MA groups. The results of cytochrome-c and BAX were concordant with enhanced autophagy of mitochondria. Qi et al. [25] showed that Bcl-2 phosphorylation and disruption of the Bcl-2/beclin-1 complex was essential to autophagy triggering and reduced mitochondrial damage after cerebral ischemia. Baek et al. [34] showed that carnosine used as a protective agent after cerebral ischemia played its beneficial roles in part through mitochondrial protection and autophagy attenuation. Nevertheless, additional study is still necessary to examine and understand the mechanisms involved in the protective effects of IpostC against I/R injury.

In summary, the present study strongly suggests that the neuroprotection induced by IpostC against I/R injury is mediated, at least in part, by promotion of autophagy during early reperfusion. The autophagy pathway may be a novel target for the development of new clinical treatments for ischemic stroke.

## Electronic supplementary material

Below is the link to the electronic supplementary material.

Figure S1. Experimental protocol used to evaluate the role of autophagy in the neuroprotective effect of ischemic postconditioning (IpostC). Middle cerebral artery occlusion (MCAO) was used as the model of cerebral ischemia-reperfusion (I/R) injury. Sham group: surgical procedure undertaken but without MCAO; I/R group: MCAO for 100 min followed by reperfusion for 1, 6, 12, 24, or 48 h; IpostC group: MCAO for 100 min, reperfusion for 10 min, MCAO for a further 10 min, then reperfusion for 1, 6, 12, 24 and 48 h; IpostC+3MA group: MCAO for 100 min, reperfusion for 10 min with 400 nM of 3-methyladenine (3MA) administered 30 min before reperfusion, MCAO for a further 10 min, then reperfusion for 24 h; and IpostC+Veh group: as negative control for the IpostC+3MA group, but with an equal volume of sterile saline administered instead of 3MA. (TIF 1485 KB)
