# Supplementary figures and images for: Ischemic Postconditioning Alleviates Cerebral Ischemia–Reperfusion Injury Through Activating Autophagy During Early Reperfusion in Rats
Source: Neurochem Res. 2018 Jul 25;43(9):1826–40. doi: 10.1007/s11064-018-2599-3 (PMC6096887; doi:10.1007/s11064-018-2599-3)

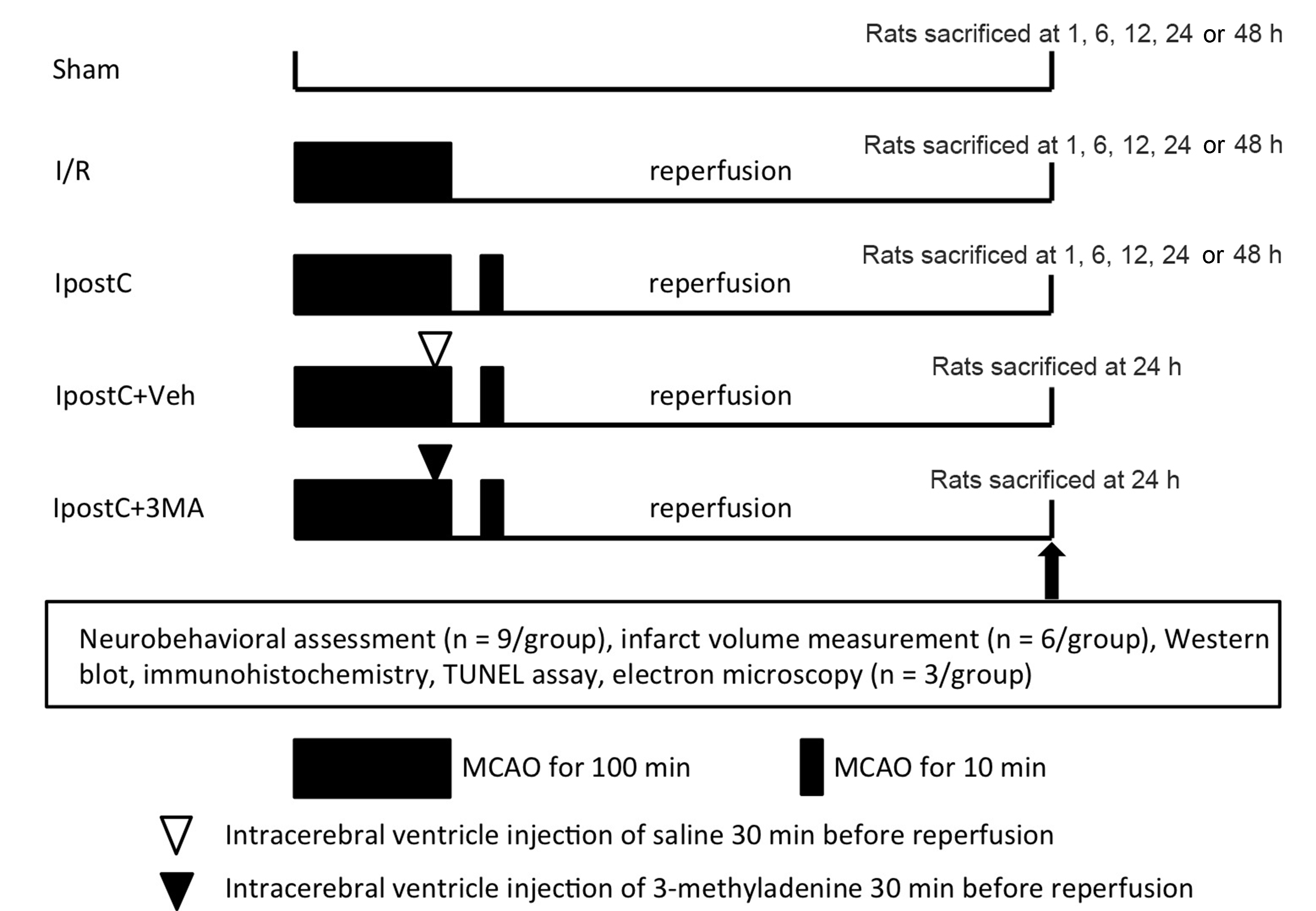

Supplement: Supplementary file 1 — Figure S1. Experimental protocol used to evaluate the role of autophagy in the neuroprotective effect of ischemic postconditioning (IpostC). Middle cerebral artery occlusion (MCAO) was used as the model of cerebral ischemia-reperfusion (I/R) injury. Sham group: surgical procedure undertaken but without MCAO; I/R group: MCAO for 100 min followed by reperfusion for 1, 6, 12, 24, or 48 h; IpostC group: MCAO for 100 min, reperfusion for 10 min, MCAO for a further 10 min, then reperfusion for 1, 6, 12, 24 and 48 h; IpostC+3MA group: MCAO for 100 min, reperfusion for 10 min with 400 nM of 3-methyladenine (3MA) administered 30 min before reperfusion, MCAO for a further 10 min, then reperfusion for 24 h; and IpostC+Veh group: as negative control for the IpostC+3MA group, but with an equal volume of sterile saline administered instead of 3MA. (TIF 1485 KB) [file 11064_2018_2599_MOESM1_ESM.tif]
